# Effect of Middle Ear Prosthesis Diameter in Platinotomy and Partial Platinectomy on Hearing Gain: A Finite Element Study

**DOI:** 10.3390/ma18133002

**Published:** 2025-06-25

**Authors:** Mario Ceddia, Nicola Quaranta, Vito Pontillo, Alessandra Murri, Alessandra Pantaleo, Bartolomeo Trentadue

**Affiliations:** 1 Department of Mechanics, Mathematics and Management, Polytechnic of Bari, 70125 Bari, Italy; bartolomeo.trentadue@poliba.it; 2 Department of Translational Biomedicine and Neuroscience, University of Bari Aldo Moro, 70125 Bari, Italy; nicola.quaranta72@gmail.com (N.Q.); alessandrapantaleo18@gmail.com (A.P.); 3 UOC Otolaryngology, University AOUC Policlinico di Bari, 70125 Bari, Italy; pontillovito@gmail.com (V.P.); alessandramurri20@gmail.com (A.M.)

**Keywords:** otosclerosis, middle ear prosthesis, prosthesis diameter, finite element analysis

## Abstract

This study investigates, for the first time, using finite element analysis (FEA), the differential impact of middle ear prosthesis diameter on hearing gain in two distinct surgical techniques: stapedotomy and partial stapedectomy. The model represented the cochlea as two fluid-filled straight channels separated by the basilar membrane and considered pistons of 0.4 mm and 0.6 mm diameters. The results demonstrated that in stapedotomy, a 0.6 mm diameter piston yielded a significantly better reduction in ABG (8.31 dB) compared to the 0.4 mm piston (10.67 dB), indicating improved hearing gain. Conversely, in partial stapedectomy, the smaller 0.4 mm piston was more effective, reducing ABG to 11.2 dB versus 12.12 dB with the larger piston. These findings highlight that the optimal prosthesis diameter varies according to surgical technique, emphasizing the need for tailored prosthesis selection.

## 1. Introduction

In patients with conductive hearing loss, the transmission of sound from the outer ear to the inner ear may be impaired [1,2,3]. A typical condition that can lead to this impairment is otosclerosis, characterized by ankylosis of the stapes (or stirrup bone) [4,5,6]. This abnormality is characterized histologically by bone deposits in the ante fenestra fissure in 80% of cases. Clinically, it is known as “Schwartze’s sign”, a reddish discoloration behind the tympanic membrane due to increased vascularization of the cochlear promontory. Progression of the disease may lead to fixation of the stapes at its base, causing transmissive hearing loss and typically closure of the air–bone interval at 2000 Hz. The histological prevalence of otosclerosis ranges from 2.5 to 8.3%, while the clinically significant prevalence is estimated to be 0.3% [1,7,8,9].

Otosclerosis primarily affects people of Caucasian ethnicity, with an incidence rate of 1%, and occurs in 0.5% of Asians. It remains one of the most common causes of conductive hearing loss in adults and is principally treated by stapedotomy or partial stapedectomy. Significant advances in diagnostic imaging over the past two decades, such as high-resolution computed tomography (HRCT) and intraoperative optical coherence tomography (iOCT), have improved the preoperative and postoperative assessment of otosclerosis. These advances allow for more accurate patient selection and more precise surgical planning [10,11].

Clinically, modern stapedotomy is frequently performed using a laser-assisted approach, with microdrills or endoscopic assistance further minimizing footplate trauma and operative time [12,13]. In partial stapedectomy, one-third or one-half of the posterior portion of the stapes is removed.

The procedure is performed by creating a hole in the footplate, followed by separating the incudostapedial joint and then removing the stapes superstructure. Microhooks are used in the removal of the desired portion of the footplate, and a prosthesis (piston) is placed into the vestibule. In stapedotomy, a small hole is made, while in partial stapedectomy, a larger portion of the footplate is removed.

Piston prostheses currently available can be broadly classified by material (metals, polymers, and ceramics) and design (shaft, loop, or hook type; self-crimping or manual crimping). A typical piston prosthesis consists of a slender piston (with a diameter of 0.4–0.8 mm), a fixation loop that engages the long process of the incus, and, when present, a collar or flange that seals the perilymphatic opening [14,15].

Titanium alloys are prevalent among metallic implants due to their low density (approximately 4.5 g/cm^3^), high biocompatibility, and Young’s modulus of approximately 110 GPa. Ultra-high-molecular-weight polyethylene (UHMWPE) and polytetrafluoroethylene (PTFE, with a density of approximately 2.2 g/cm^3^ and an elastic modulus of approximately 0.5 GPa) are easily customized via injection molding [16,17,18,19].

Ceramic prostheses, which are commonly hydroxyapatite-based with a density of ≈3.1 g/cm^3^ and a Young’s modulus of ≈100 GPa, are fabricated by powder sintering or ceramic injection molding. They provide excellent stiffness but have less intraoperative flexibility. Critical mechanical parameters, such as compressive strength (titanium > 100 MPa, ceramic ≈ 60 MPa, and polytetrafluoroethylene (PTFE) ≈ 40 MPa) and flexural strength (titanium > 200 MPa, ceramic ≈ 150 MPa, and polymers 30–50 MPa), directly impact vibrational transmission and long-term stability [20,21,22,23].

Manufacturing techniques for all of the above-mentioned differ markedly. Titanium prostheses are usually laser-cut from sheet metal or machined using computer numerical control (CNC), though some centers use selective laser melting (SLM) for custom designs. Ceramics require high-temperature sintering and precision machining, while polymers use cost-effective injection molding and extrusion processes. These processes also facilitate the incorporation of antimicrobial additives, such as silver nanoparticles, to reduce postoperative infection [24,25].

The choice of closure material, such as fat, fascia, or synthetic sealants, further modifies the effective stiffness of the reconstructed ossicular chain. Studies have shown that less rigid materials improve early postoperative hearing outcomes [26].

The numerical simulation of middle ear behavior has evolved rapidly and now plays a central role in prosthesis design. While early finite element models only addressed the ossicular chain and the tympanic membrane, recent studies have incorporated the anisotropic, frequency-dependent properties of bone and soft tissue, as well as fluid–structure interactions within the cochlea [27,28,29,30]. Liang et al. [31] developed a multiphysics finite element analysis (FEA) model of the middle ear, including the tympanic membrane, ossicular chain, and cochlear fluids. They demonstrated that millimeter-scale changes in piston geometry can significantly alter the harmonic response up to 8 kHz. Caminos et al. [32] extended this approach by implementing anisotropic material properties for bone and soft tissue. This highlighted critical stress concentrations on the stapes during virtual stapedotomy. Using a compressible fluid model, Alimi et al. [33] evaluated how perilymph viscosity influences sound transmission and correlated basilar membrane vibration peaks with piston diameter. A study conducted by Ataide et al. [34] compared titanium, polytetrafluoroethylene (PTFE), and ceramic pistons, quantifying the trade-off between stiffness and acoustic transmission. Differently from other studies, Asakura et al. [35] applied topology optimization within the finite element analysis (FEA) framework to suggest variable section piston designs that minimize incus stress while maintaining high acoustic efficiency. Multiphysics finite element method (FEM) platforms now enable virtual prototyping under physiological loading. These platforms can predict both static displacement and harmonic response up to 10 kHz and guide optimization of piston diameter, length, and material to maximize cochlear stimulation while avoiding stress concentrations [36,37,38].

Although existing studies have analyzed how prosthesis diameter affects hearing outcomes in stapes surgery, these analyses have mostly focused separately on stapedotomy and stapedectomy, without a direct and systematic comparison of prosthesis size effects between the two procedures. This study uses a detailed finite element method (FEM) model of fluid–structure interaction to evaluate the improvement in hearing calculated using the air–bone gap (ABG) parameter. The model is made of polytetrafluoroethylene (PTFE) with two different diameters (0.4 and 0.6 mm) and is used in stapedotomy and partial stapedectomy procedures.

## 2. Materials and Methods

The three-dimensional geometry of the middle ear and cochlea model was designed using Autodesk Inventor 2024, which allowed the creation of a detailed and accurate CAD model of the anatomical structures involved. The model consists of two straight canals that represent the cochlea.

**Figure 1:**
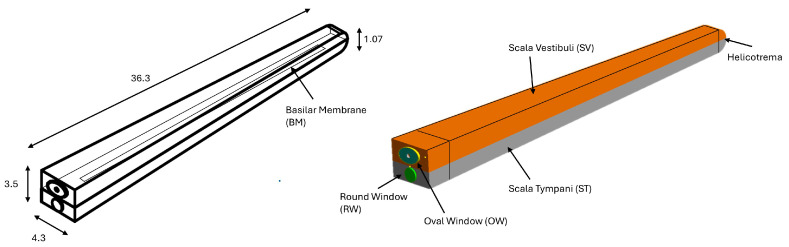
Simplified cochlea model structures with anatomical parts and dimensions.

Other FE models, such as those of Areias et al. [39], Zhang X, and Zhang C.S. [40,41], represent the cochlea as a spiral structure to improve anatomical accuracy. However, these complex models do not offer significant advantages for the purposes of this research. Therefore, the three-dimensional finite element model of the uncoiled cochlea has been constructed as two straight fluid-filled channels, representing the scala vestibuli (SV) and the scala tympani (ST), separated by the basilar membrane (BM) (see Figure 1). The base of the stapes is anchored to the SV at the oval window via an annular ligament. Both channels, SV and ST, are filled with a viscous fluid known as perilymph and are interconnected at the apical regions through the helicotrema. The geometry of the inner ear structures has been derived in accordance with the dimensions reported by Kwacz et al. [42].

**Figure 2:**
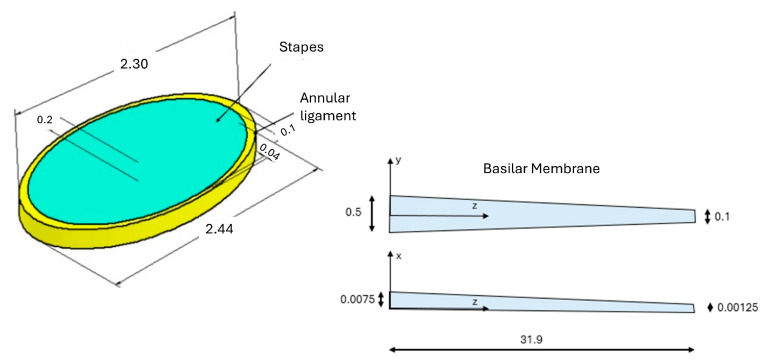
Simplified stapes model structures with anatomical parts and dimensions.

The base of the stapes is modeled as an ellipse with a major axis of 2.24 mm and a minor axis of 1.4 mm. The annular ligament is represented as an elliptical ring, with a width varying from 0.1 mm to 0.04 mm. Both the annular ligament and stapes have a thickness of 0.2 mm. The round window membrane (RW) is modeled as a circle with a diameter of 1.2 mm and a thickness of 0.05 mm. All nodes on the outer perimeters of the annular ligament and round window membrane are fixed, while the inner nodes of the annular ligament are connected to the outer nodes of the stapes Figure 2 [42,43].

### 2.1. Geometric Models for Stapedotomy and Partial Stapedectomy

**Figure 3:**
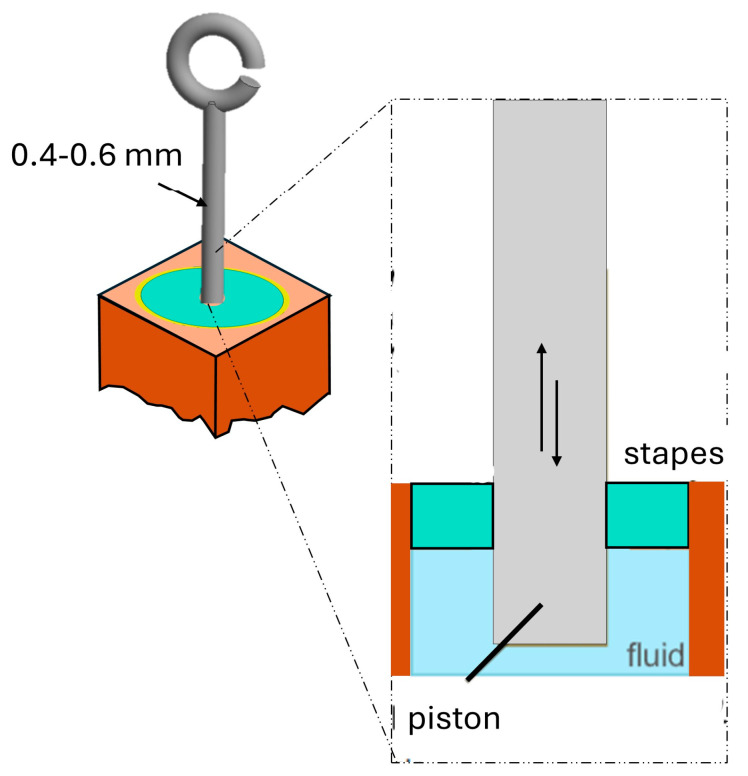
Representation of the piston inserted into the hole in the stapes.

**Figure 4:**
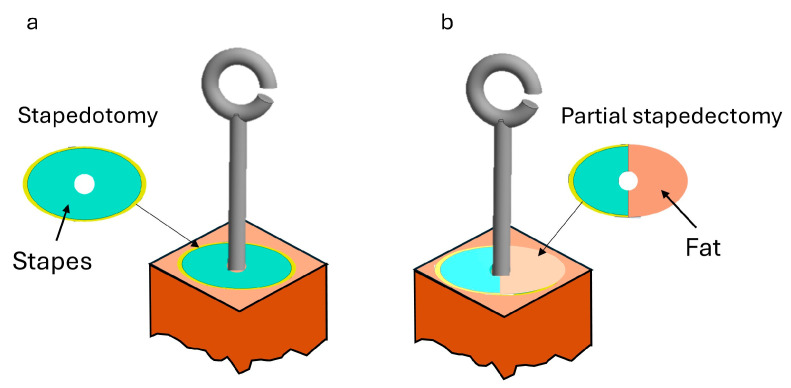
Details of the models analyzed. (**a**) stapedotomy model; (**b**) partial stapedectomy model.

In the stapedotomy model, a small 0.4 or 0.6 mm hole is created in the center of the stapes, which otherwise remains intact (Figure 3). As shown in Figure 4a, the piston is inserted directly into the vestibular cavity. The piston matches the diameter of the hole and extends 0.30 mm beyond the footplate. In the partial stapedectomy model, approximately half of the stapes are surgically removed and substituted with autologous fat Figure 4b. The same piston (with diameters of 0.4 and 0.6 mm and a 0.30 mm extension) is inserted into the vestibule through the enlarged opening.

### 2.2. Materials

The material properties of the inner ear structures in the finite element model were derived from the literature [27,44,45]. The characteristics of each structure, including density, Young’s modulus, and Poisson’s ratio, are written in detail in Table 1. Most of the solid materials were treated as isotropic, and the perilymph fluid was modeled as a viscous fluid with a density of 1000 kg/m^3^ and a viscosity of 0.001 Ns/m [42,46,47].

### 2.3. Analytical Evaluation of the Air Conduction (AC)

To evaluate the hearing gain expressed by the air–bone gap (ABG) value, the air conduction (AC) value was first assessed. The middle ear was modeled using its equivalent impedance, as derived from studies conducted by Rosowsky et al. [48,49] and Mason et al. [50]. Equation (1) is as follows:

(1) $$Z_{MH}=Z_{C}\cdot \left(\frac{A_{TM}}{A_{FT}}\right){\left(\frac{M_{L}}{LIL}\right)}^{2}$$

where:

Z_MH_ = impedance of the healthy middle ear [Pa·s/m]

Z_C_ = cochlear impedance [Pa·s/m]

A_TM_ = area of the tympanic membrane (eardrum) [mm^2^]

A_FT_ = area of the stapes footplate [mm^2^]

M_L_ = length of the malleus manubrium (lever arm of malleus) [mm]

L_IL_ = length of the incudostapedial lever [mm]

M_L_/L_IL_ = amplification ratio along the ossicular chain (manubrium/incus and incus/stapes)

Using the corresponding values from a study conducted by Mason et al. [50], a healthy ear has an equivalent impedance of 236.686 [Pa·s/m]. However, the presence of otosclerosis leads to an increase in the equivalent impedance (Z_OT_), which can be calculated using the following Equation (2):

(2) $$Z_{OT}=Z_{MH}\cdot \left(1+\frac{AC}{10}\cdot 0.25\right)$$

where:

Z_OT_ = equivalent impedance in the presence of otosclerosis [Pa·s/m]

Z_MH_ = equivalent impedances healthy ear [Pa·s/m]

AC = Air conduction [dB]

Therefore, from Equation (2), AC can be evaluated as Equation (3):

(3) $$AC=\left(\frac{Z_{OT}}{Z_{MH}}-1\right)\cdot \frac{10}{0.25}$$

The impedance in the presence of otosclerosis (Z_OT_) can also be calculated as a function of sound pressure (P), the vibrating surface area (A), frequency (f), and the displacement (d) of the stapes or piston. The evaluation is conducted via finite element analysis (FEA), with the governing formula (Equation (4)) reworked on the basis of the studies by Koike et al. [44] and Withnell et al. [51]:

(4) $$Z_{OT}=\frac{P}{f\cdot d}\cdot A$$

For the FEA analysis, a pressure of 1.25 Pa, equivalent to a sound pressure level of 95 dB [50,51], is considered. Meanwhile, the lower site of the piston is studied in the same manner as the area of the vibrating surface.

### 2.4. FEA Modeling

**Figure 5:**
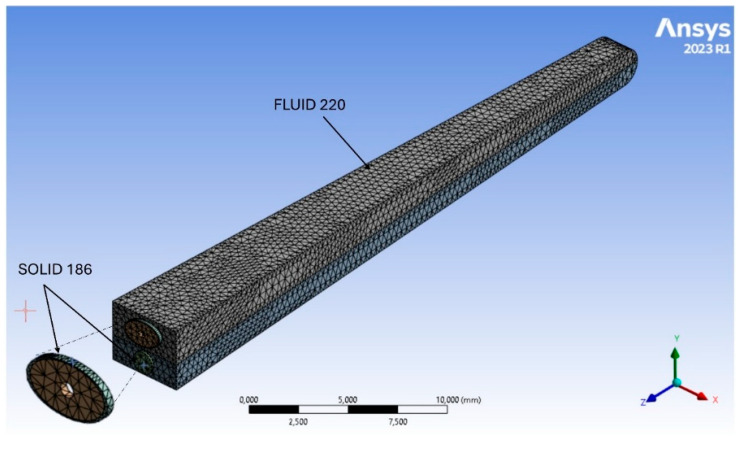
Mesh modeling of the anatomical structure of the middle ear.

The FEA model was analyzed using Ansys R 2023 software, with the spiral canal of the cochlea modeled as a two-channel untwisted system consisting of 34,217 quadratic acoustic fluid elements (FLUID 220) with a compressible formulation. The scala vestibuli, scala tympani, and vestibulum are finely meshed to ensure accurate sound wave propagation. Outer walls of the fluid compartments are modeled as rigid, while fluid–structure interfaces enable dynamic coupling with all types of adjacent solid membranes, particularly the basilar membrane. Using a compressible formulation effectively captures high-frequency acoustic resonance phenomena, enhancing the model’s physical accuracy compared to incompressible approaches [52,53]. The basilar membrane (BM), which separates the two scales, has been modeled as a thin volume composed of 4517 quadratic solid elements (SOLID 186), with elements distributed along the thickness of the membrane. The prosthesis piston was meshed using 1034 quadratic tetrahedral solid elements (SOLID 187), as shown in Figure 5. All contact faces between the solid bodies and fluid have a fluid–structural interface. To ensure a consistent mesh, a shared topology is employed between the piston and the vestibule, ensuring that the nodes of the fluid structures coincide and are shared with those of the prosthesis. The piston is naturally constrained in the footplate (xz-plane) by the fluid’s inertia [42,53]. The interaction between the basilar membrane (BM) volume and the volumes of the scala vestibuli (SV) and scala tympani (ST) in the FE model was addressed by defining fluid–structure interfaces, which allow the transfer of forces and vibrations between the solid membrane and the fluids in the two scalae. A dynamic finite element analysis coupled with fluid–structure interaction was performed to simulate the acoustic vibration of the system.

## 3. Results

### 3.1. Validation of the FE Model

The finite element (FE) model of the normal cochlea analyzed in this study was validated by comparing the displacement–frequency curves of the stapes with experimental data from a study conducted by Burovikhin et al. [43]. Through the study, a coupled finite element model of the middle and inner ear was developed and validated. This model is capable of simulating sound transmission and evaluating the performance of stapes prostheses. This virtual model allows one to analyze the effects that different prostheses have on the human ear.

**Figure 6:**
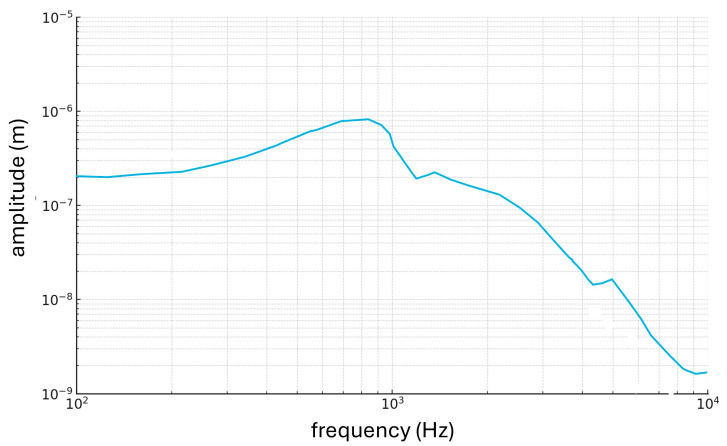
Displacement of the stapes as frequencies change.

Figure 6 shows that the maximum peak displacement of the stapedial plate occurs at a frequency of 750 Hz rather than 1000 Hz, the typical frequency of a healthy ear. This variation in the peak suggests that the pathological condition, such as otosclerosis, has a significant effect on sound transmission through the middle ear [54]. In addition, it is observed that the curve describing the relationship between stapes displacement and frequency tends to decrease as the latter increases. This phenomenon can be explained by the fact that at higher frequencies, the stiffness of the ossicular chain becomes a predominant factor limiting the efficiency of sound transmission. The increase in stiffness at higher frequencies helps to dampen the vibrations of the stapedial plate, thereby reducing the amplitude of the vibrations.

### 3.2. Determination of Piston Displacements in Stapedotomy and Partial Stapedectomy

**Figure 7:**
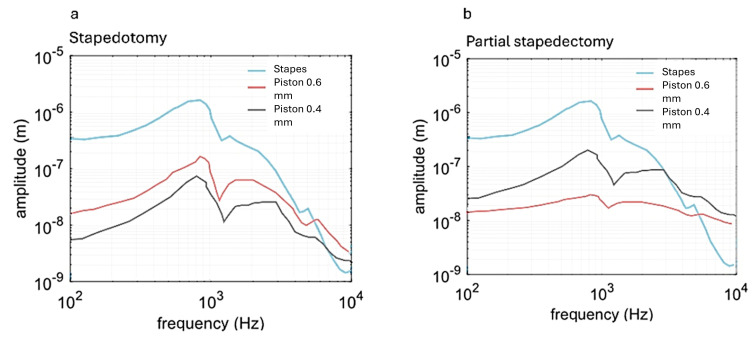
Displacements of the piston with varying frequency. (**a**) stapedotomy; (**b**) partial stapedectomy.

After validation of the FEM model, the piston base displacements were evaluated in a frequency analysis. Figure 7 compares the two curves for stapedotomy and partial stapedectomy using two pistons with different diameters (0.4 mm and 0.6 mm).

In stapedotomy, the 0.4 mm piston produced a maximum displacement of 2.34 × 10^−7^ m at 980 Hz, while the 0.6 mm piston peaked at 2.85 × 10^−8^ m at 750 Hz (Figure 7a). In partial stapedectomy, the 0.4 mm piston reached 2.75 × 10^−8^ m at 682 Hz, compared with 1.67 × 10^−8^ m for the 0.6 mm piston at the same frequency (Figure 7b). The larger piston diameter in stapedotomy provides a greater surface area for coupling to the perilymph, yielding higher vibration amplitudes when maximal sound wave transmission is required. By contrast, in partial stapedectomy, where residual ossicular linkages contribute to the movement, a smaller piston minimizes inertial loading and, thus, more efficiently transmits vibrations under these conditions.

### 3.3. Calculation of ABG in Stapedotomy and Partial Stapedectomy

From the frequency analysis results, the piston displacements in both stapedotomy and partial stapedectomy were calculated at the following frequencies: 500 Hz, 1000 Hz, 2000 Hz, and 4000 Hz, as reported in Table 2.

**Figure 8:**
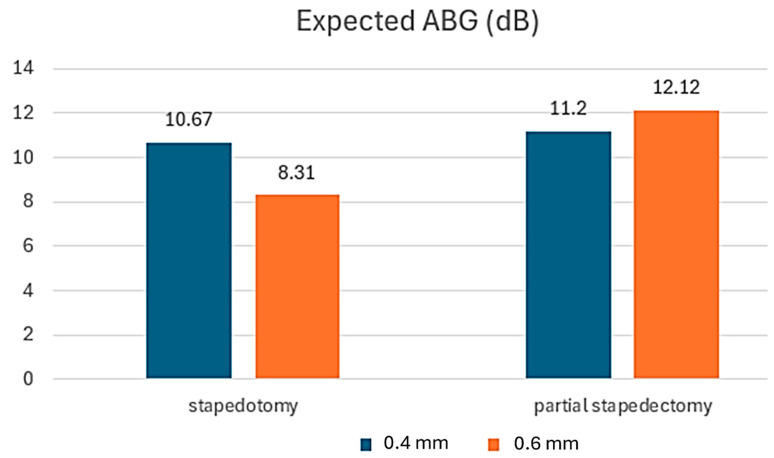
Results of the average ABG for the two pistons (0.4 mm and 0.6 mm) in stapedotomy and partial stapedectomy.

The air–bone gap (ABG) was obtained by subtracting bone conduction (BC) from air conduction (AC) [55]. AC was determined using Equation (3), averaging the values at 0.5, 1, 2, and 3 kHz. In the stapedotomy group, AC was 36.57 dB with the 0.4 mm piston and 34.21 dB with the 0.6 mm piston. In the partial stapedectomy group, AC was 37.10 dB with the 0.4 mm piston and 38.02 dB with the 0.6 mm piston. The bone conduction (BC) values were obtained from a study conducted by Quaranta et al. [55]. Since our numerical model was unable to reproduce Carhart’s clinical effect, we assumed that the BC values remained unchanged after surgery [43]. Figure 8 illustrates the ABG values calculated for stapedotomy and partial stapedectomy as a function of the two implant diameters.

The analysis of results obtained from stapedotomy and partial stapedectomy procedures reveals a notable relationship between piston diameter and changes in the air–bone gap (ABG). In stapedotomy, the use of a 0.6 mm diameter piston leads to a reduction in the ABG to 8.31 dB, compared to 10.67 dB observed with a 0.4 mm piston. This indicates enhanced auditory gain associated with the larger piston size. Conversely, in partial stapedectomy, optimal results are achieved with the smaller piston diameter. Here, the ABG reaches 11.2 dB with a 0.4 mm piston, whereas it increases to 12.12 dB when using a 0.6 mm piston. These findings suggest that, within the context of partial stapedectomy, a smaller piston offers superior effectiveness in improving sound transmission compared to a larger piston. In summary, the results demonstrate that a larger diameter piston in stapedotomy promotes ABG reduction and consequently improves hearing, while in partial stapedectomy, a smaller piston diameter yields better auditory outcomes. These observations have important implications for selecting the appropriate piston size based on the surgical procedure performed.

## 4. Discussion

Both stapedotomy and partial stapedectomy surgical techniques have their proponents, but there has been a recent trend favoring stapedotomy [55,56,57]. This is likely due to studies demonstrating more effective mechanical sound transmission and reduced risk to the vestibular and auditory systems [58,59,60]. Several investigations have reported superior auditory outcomes with stapedotomy compared to partial stapedectomy, concluding that stapedotomy more effectively closes the air–bone gap (ABG), with fewer complications and greater technical ease [26,54,61,62,63]. In contrast, Fisch et al. [54] analyzed 340 cases divided into total/partial stapedectomy and stapedotomy groups and found no significant differences in ABG across frequencies of 0.5–2 kHz over follow-up periods ranging from 3 weeks to 3 years. Grégorie et al. [64] conducted a statistical analysis of 1681 otosclerosis cases involving stapedotomy, accounting for 85% of the total cases. At the six- to twelve-month follow-up, the stapedotomy group demonstrated significantly greater postoperative improvement in air conduction at 4 kHz than the partial and total stapedectomy groups. There was a greater improvement at 8 kHz with the total stapedectomy group. In a randomized study of 100 patients, Bailey et al. [65] observed significant short-term improvements in air conduction after surgery at 2, 4, and 8 kHz in favor of stapedotomy. Moon et al. [66], in a study of 106 stapedectomy cases (defined as >50% footplate removal) and 264 stapedotomy cases, reported significantly better air conduction gain at 2, 4, and 8 kHz after 6 months in the stapedotomy group. In contrast, Quaranta et al. [55] compared 72 partial stapedectomies with 79 stapedotomies and found no statistically significant difference in the percentage of ABG closure after 6 weeks of follow-up. These mixed results highlight ongoing debate but suggest a trend toward improved auditory outcomes with stapedotomy in several studies.

Dhooge et al. [67] compared 60 stapedectomies with 55 stapedotomies, demonstrating superior outcomes in the stapedotomy group, particularly in the high-frequency range. Nonetheless, auditory gain results depend not only on the surgical technique but also on factors related to prosthesis geometry. Over the years, various pistons with diameters ranging from 0.3 to 0.8 mm have been developed to optimize postoperative hearing outcomes. Mathematical models and temporal bone studies have indicated that using a larger-diameter piston enhances acoustic transmission and, consequently, improves postoperative pure-tone audiometry (PTA) results. However, smaller diameter pistons are thought to reduce the risk of iatrogenic trauma and subsequent sensorineural hearing loss (SNHL) [15,57,68]. Several studies have reported significant differences in audiometric outcomes favoring the 0.6 mm piston [15,69,70]. Conversely, Rompaey et al. [71] found a higher proportion of cases achieving an air–bone gap closure of 10 dB or less in groups fitted with 0.3 mm and 0.5 mm pistons (56% and 60%, respectively) compared to those with 0.4 mm and 0.6 mm pistons (32% and 33%). Additionally, Grolman et al. [72] compared Teflon pistons of 0.3 mm and 0.4 mm diameters, demonstrating clinically relevant differences in air conduction gain (5–11 dB) at low frequencies (125, 250, 500, and 1000 Hz) favoring the 0.4 mm piston. These studies highlight the presence of controversial results regarding the use of smaller or larger diameter pistons. In addition, these analyses did not consider the impact of piston diameter on the two surgical techniques, stapedotomy and partial stapedectomy. This study, conducted using the finite element method, showed that the optimal results in terms of air–bone gap (ABG) are obtained with a piston of 0.6 mm in stapedotomy and with a piston of 0.4 mm in stapedectomy. Similarly, in a study conducted by Burovikhin et al. [43], increasing the piston diameter in stapedotomy from 0.6 mm to 0.7 mm resulted in an improvement in hearing performance of about 1 dB.

### Limitations

Finite element analysis (FEA) is a valuable tool for simulating inner ear dynamics and optimizing stapes prostheses; however, several limitations must be acknowledged [73,74,75,76]. Firstly, modeling often involves simplifications of the intricate anatomy of the inner ear, which can compromise predictive accuracy and reduce the ability to faithfully replicate physiological conditions. Additionally, current models commonly assume a piston-like motion for the stapes footplate, whereas actual physiology may involve high-frequency oscillatory movements. Neglecting these mechanisms can limit understanding of sound transmission. Another challenge lies in representing the anisotropic material properties of biological tissues, which complicate modeling and can significantly affect simulation outcomes. FEA also relies on high-quality experimental data for validation, but the limited availability of such data, particularly for various pathological conditions, hinders accurate model verification. Moreover, complex biological interactions, such as the sealing around the prosthesis and fluid–tissue interactions, may not be fully integrated into FEA models, restricting insight into their impact on prosthesis performance. Finally, the computational intensity of FEA, especially for detailed three-dimensional models, can constrain the ability to perform comprehensive simulations or to implement effective optimization strategies in prosthesis design. Additionally, our current model does not simulate bone conduction and, therefore, cannot predict outcomes associated with its variation, such as postoperative recovery of the Carhart notch. In future work, we intend to incorporate this effect through more accurate modeling that directly correlates numerical simulations with clinical data. Addressing these limitations is essential for advancing the application of FEA in otologic research and prosthetic development.

## 5. Conclusions

The results of the study demonstrated a significant relationship between the diameter of the prosthetic piston and the air–bone gap (ABG) in stapedotomy and partial stapedectomy procedures. However, the dimensional assessment of the piston must always be contextualized with respect to the material used, as the resulting stiffness depends not only on the diameter of the prosthesis but also on the mechanical properties of the material. For instance, the use of Teflon, characterized by appropriate flexibility and biocompatibility, allows for effective sound transmission without compromising the integrity of surrounding tissues. Specifically, in stapedotomy, a 0.6 mm diameter piston resulted in a lower ABG (8.31 dB), indicating better hearing improvement compared to the 0.4 mm piston (10.42 dB). In contrast, for partial stapedectomy, the 0.4 mm piston proved more effective, achieving an ABG of 10.67 dB as opposed to 12.12 dB with the 0.6 mm piston. These findings suggest that the optimal piston size may vary depending on the surgical technique and the material properties of the prosthesis, highlighting the importance of a tailored approach to prosthesis selection based on both clinical and mechanical considerations. Further research is needed to confirm these findings and to investigate more closely the influence of prosthesis geometry and material composition on auditory outcomes.

## Figures and Tables

**Table 1:** Mechanical properties of the ear structures and prosthetics used in the FEM model.

Structure	Data Used in the FEM Model
Stapes footplate Density (kg/cm^3^) Young’s modulus (GPa) Poisson’s ratio	2.30 17.1 0.3
Annular ligament Density (kg/cm^3^) Young’s modulus (GPa) Poisson’s ratio	1.20 0.0002 0.3
Implant (PTFE) Density (kg/cm^3^) Young’s modulus (GPa) Poisson’s ratio	2.2 0.575 0.3
Fat Density (kg/cm^3^) Young’s modulus (GPa) Poisson’s ratio	1.20 1 × 10^−6^ 0.3

**Table 2:** Piston displacements in stapedotomy and partial stapedectomy.

Frequency (Hz)	Stapedotomy		Partial Stapedectomy	
	Piston Diameter		Piston Diameter	
	0.4 mm	0.6 mm	0.4 mm	0.6 mm
500	2.45 × 10^−9^	2.85 × 10^−8^	2.81 × 10^−8^	1.67 × 10^−8^
1000	1.34 × 10^−8^	2.12 × 10^−8^	2.26 × 10^−8^	1.15 × 10^−8^
2000	2.23 × 10^−8^	2.45 × 10^−8^	2.48 × 10^−8^	1.21 × 10^−8^
4000	1.26 × 10^−8^	1.75 × 10^−8^	1.92 × 10^−8^	1.13 × 10^−8^
	Piston displacement (m)			

## Data Availability

The original contributions presented in this study are included in the article. Further inquiries can be directed to the corresponding author.
